# Benign multicystic peritoneal mesothelioma occurring in bilateral inguinal canals metachronously: a case report

**DOI:** 10.1186/s40792-022-01399-5

**Published:** 2022-03-16

**Authors:** Hiroyuki Oshikiri, Yohei Ozawa, On Suzuki, Masahiro Usuda, Go Miyata

**Affiliations:** 1 Department of Digestive Surgery, Iwate Prefectural Central Hospital, 1-4-1, Ueda, Morioka, Iwate 020-0066 Japan; 2 Department of Surgery, Tohoku University Graduate School of Medicine, 1-1, Seiryo-machi, Aoba-ku, Sendai, 980-8574 Japan

**Keywords:** Benign multicystic peritoneal mesothelioma, Metachronous bilateral inguinal tumor, Inguinal nodule, Immunohistochemistry, Calretinin, D2-40

## Abstract

**Background:**

Benign multicystic peritoneal mesothelioma (BMPM) is a benign tumor that usually occurs in middle-aged females. Although several published studies have reported the occurrence of this tumor in the abdominal cavity, few have documented its development in the inguinal region.

**Case presentation:**

We present a case of a 48-year-old female presenting with a bulge in her left inguinal region. Physical examination revealed a golf ball-sized nodule in the left inguinal region that could not be pushed back into the abdominal cavity. Contrast-enhanced computed tomography showed a multicystic tumor; therefore, the patient was diagnosed with inguinal hernia or hydrocele of the Nuck’s canal. We performed surgical resection and hernia repair using the mesh plug method. The resected specimen was 80 mm in length and contained a multicystic tumor. Pathological examination showed that the cyst wall was lined by a single layer of cuboidal to single layer squamous epithelium. Immunohistochemistry revealed positivity for calretinin in the epithelial cells, for which a diagnosis of BMPM was established. The patient returned to our hospital after 5 years with symptoms similar to the previous episode, but this time in the right inguinal region. Imaging studies showed a tumor in the right inguinal region with the same characteristics as the previous one. The patient underwent tumor resection and hernia repair using the same technique. The resected tumor was 45 mm in length and had characteristics similar to the previously resected tumor. The presence of calretinin and D2-40 on immunohistochemistry led to the diagnosis of BMPM. There was no recurrence of BMPM for 33 months after the secondary surgery.

**Conclusions:**

Here we present the first report of metachronous BMPM occurring in bilateral inguinal canals. Although the pathogenesis of BMPM remains unclear, reactive changes have been suggested to cause tumors originating from the groin. The treatment of choice for BMPM is surgical resection. For diagnosis, pathological examination with immunostaining can be useful. The most appropriate postoperative follow-up for inguinal BMPM is controversial, and the accumulation of more inguinal BMPM cases is needed.

## Background

Mesotheliomas are neoplasms that arise from the mesothelium, such as the pleura, peritoneum, and pericardium, with peritoneal mesothelioma accounting for less than 20% of all cases. Peritoneal mesothelioma is generally identified as diffuse malignant type histologically; however, benign multicystic peritoneal mesothelioma is diagnosed in 3–5% of cases (BMPM). Mennemeyer et al. were the first to report BMPM in 1979 [1]. Studies have reported that BMPM mainly arises in young to middle-aged females at an average age of 37 years [2], whereas malignant peritoneal mesothelioma mostly affects elderly males in their fifth and sixth decades [3]. Considering that BMPM is often found in the pelvis, differentiating it from other neoplastic diseases, such as endometriotic cysts or pseudomyxoma peritonei, has been difficult. Only a few cases of BMPM have been reported in the groin among the less than 200 previously reported cases [4]. This is the first case with metachronous BMPM in bilateral inguinal canals, according to our review of the literature.

## Case presentation

**Fig. 1:**
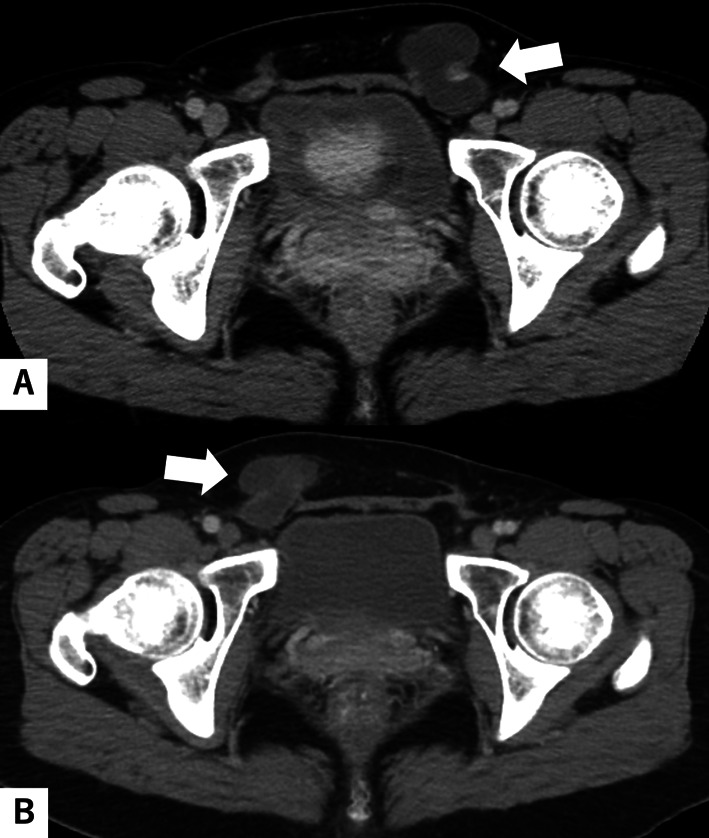
**A** Enhanced computed tomography (CT) images before the first surgery revealing multicystic lesions, suggesting a hydrocele of Nuck’s canal or inguinal hernia (arrow). No lesion was observed in the right inguinal area. **B** Enhanced CT images before the secondary surgery showing a multicystic lesion, suggesting a hydrocele of Nuck’s canal or a cystic tumor similar to that resected previously (arrow). No recurrence was noted in the left inguinal area

A 48-year-old female was referred to our hospital for a bulge in her left inguinal region. She had been aware of a nodule in her left groin for a year. She was a teacher and had no notable medical history. A subsequent medical examination revealed a golf ball-sized nodule in the left inguinal region that could not be pushed back into the abdominal cavity. Blood chemistry findings were unremarkable. Contrast-enhanced computed tomography (CT) revealed a multicystic tumor in the same region and the tumor was 38 mm in diameter. Bilateral normal ovaries were found in the pelvis with no ascites (Fig. 1A).

Owing to the difficulty in differentiating BMPM from hernia and hydrocele of Nuck’s canal, we decided to perform surgery for obtaining a diagnosis and treating the lesion. The surgery was performed via the anterior approach. After complete resection, the multicystic tumor was determined to have originated from the end of the hernia sac in the medial inguinal ring. The hernia was repaired using the mesh plug technique, and the patient was discharged without any complications.

**Fig. 2:**
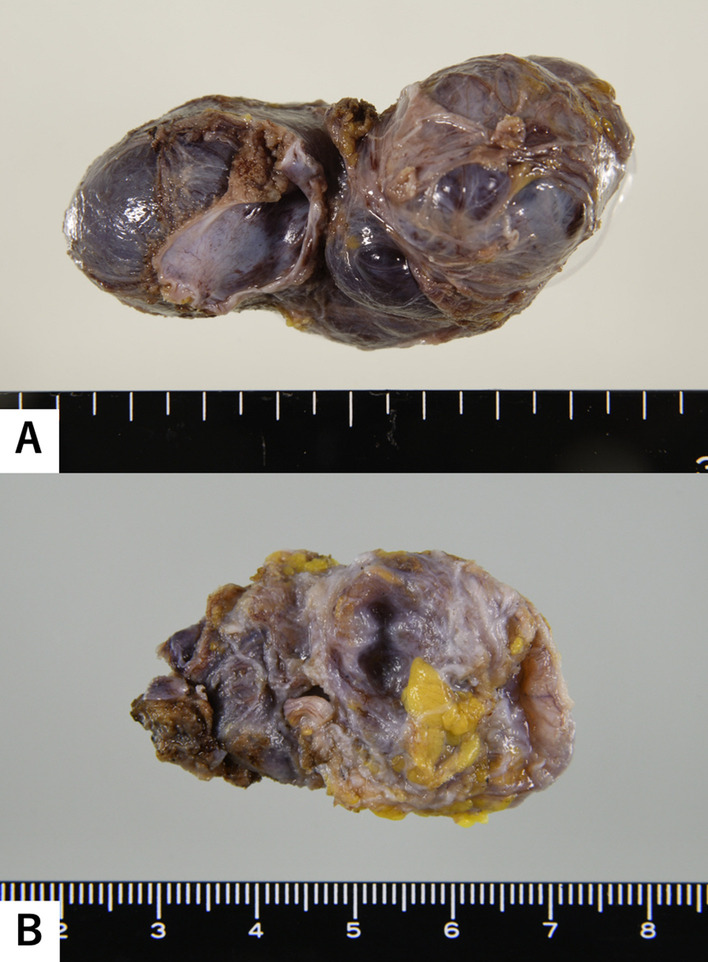
**A** Macroscopic image of the left inguinal tumor resected during the first surgery. The multicystic tumor was 80 mm in length and 35 mm in diameter and filled with serous fluid. **B** Macroscopic image of the right inguinal tumor resected during the secondary surgery. The tumor filled with serous fluid was 45 mm in length and 25 mm in diameter

**Fig. 3:**
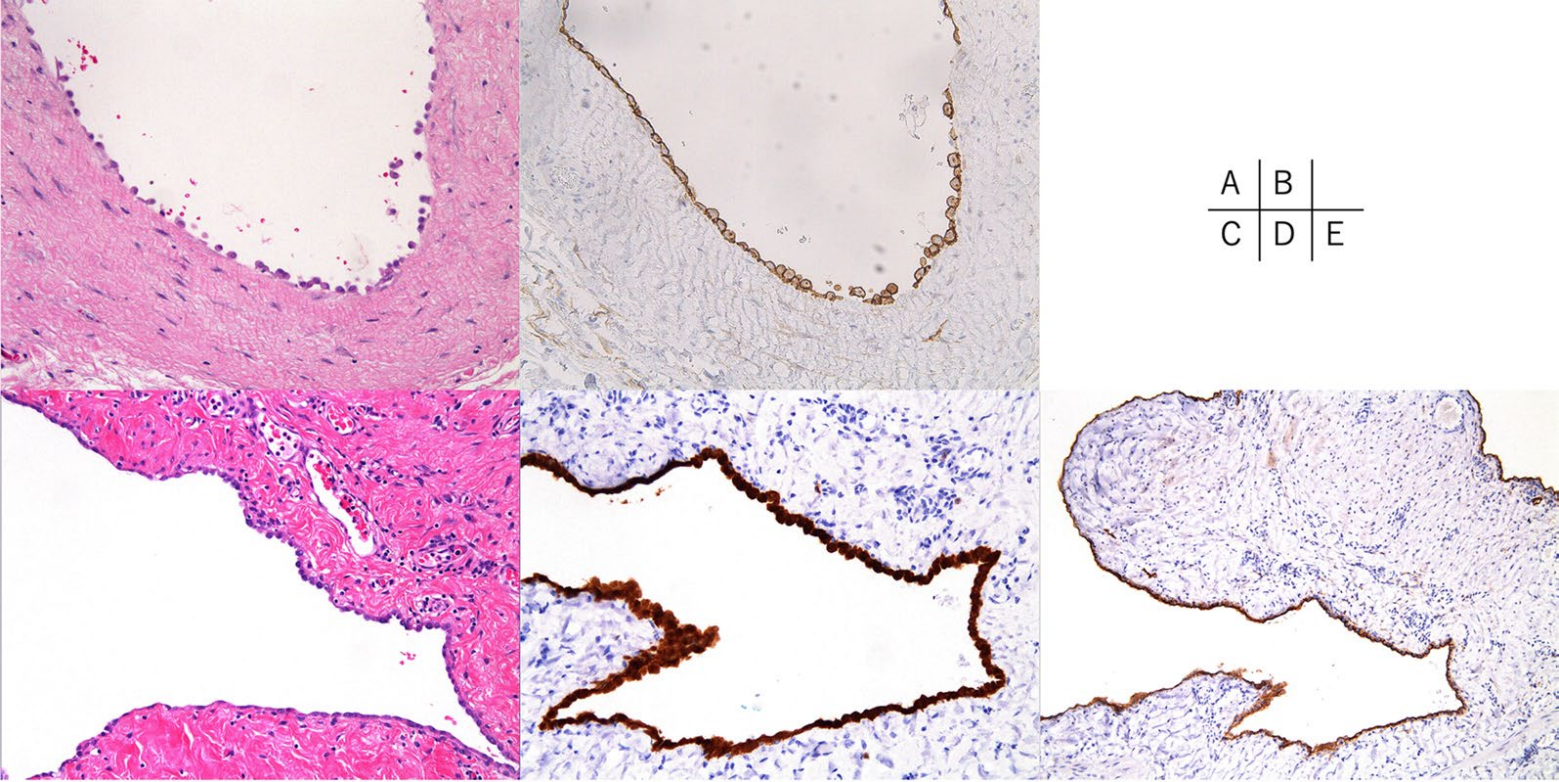
Microscopic images of the surgical specimens. **A** The cyst wall was lined by a single layer of cuboidal to single layer squamous epithelium in the first surgery (hematoxylin and eosin, ×200). **B** Calretinin immunohistochemical staining (×200) revealing a positive reaction. **C** Resected specimen in the secondary surgery showing the same findings as in a (hematoxylin and eosin, ×200). **D** Calretinin immunohistochemical staining (×200) revealing a positive reaction. **E** Immunohistochemical staining also showed D2-40 positivity (×100)

The surgically resected specimen contained a multicystic tumor at the end of the hernia sac that was 80 mm in length and 35 mm in diameter. No substantial components were found in the cyst. The inside of the cyst was filled with serous fluid (Fig. 2A). Pathological examination revealed that the cyst wall was lined by a single layer of cuboidal to single layer squamous epithelium. No cellular or structural atypia was observed. The tumor, which showed positivity for calretinin in the epithelial cells, was finally diagnosed as BMPM by immunohistochemistry (Fig. 3A, B).

The patient was followed up after the first postoperative visit. However, the patient returned to our hospital 5 years later with similar symptoms contralaterally but in the right inguinal region this time. Abdominal contrast-enhanced CT revealed fluid collection in the right inguinal canal and the size of the content was 27 mm in diameter (Fig. 1B). No recurrence was noted in the left inguinal region or any other intra-abdominal space; we suspected a hydrocele of Nuck’s canal or cystic tumor, such as BMPM. Therefore, the tumor was resected and an inguinal hernia was repaired using the mesh plug technique in a procedure similar to the previous one.

The resected specimen contained a multicystic tumor at the end of hernia sac that was 45 mm in length and 25 mm in diameter. Macroscopic findings were very similar to the previously resected tumor (Fig. 2B). Moreover, pathological findings showed characteristics similar to the previous, with epithelial cells being positive for calretinin and D2-40 following immunostaining. Therefore, the tumor was diagnosed as BMPM. After surgery, the patient was discharged with no complications, and no tumor recurrence was found for 33 months.

## Discussion

Benign multicystic peritoneal mesothelioma (BMPM) is generally considered a peritoneal reaction secondary to chronic irritation with mesothelial cell entrapment, reactive proliferation, and cystic formation [5]. Studies have shown that 30–87% of patients who developed BMPM had previously undergone abdominal surgery [4]. Moreover, chronic inflammation, endometriosis, and peritonitis related to peritoneal dialysis have also been reported to be involved in the development of BMPM [6]. In addition, some reports have found that cystic tumors are formed when ovarian fluid is trapped in peritoneal adhesions or inflammation [7]. However, other reports consider BMPM to be an oncological occurrence. Despite having no history of abdominal surgery, some BMPM patients exhibit neoplastic growth, according to Weiss et al., supporting the theory of neoplastic change [8]. Two BMPMs occurred metachronously at different sites (bilateral inguinal canals) in our case, with no other recurrences. Due to the lack of a direct vascular and lymphatic connection between the two inguinal canals, it seems unlikely that the two lesions were caused by the same source. Six cases of inguinal BMPM (Table 1) showed no recurrence after surgical resection [9–14], whereas one case involving an elderly man developed postoperative recurrence [15]. Chronic peritoneal inflammation caused by an inguinal hernia could have caused BMPM. We believe that BMPM occurred heterotopically in the inguinal region via the aforementioned mechanism in the current case.

**Table 1:** Cases diagnosed as benign multicystic peritoneal mesothelioma in the inguinal lesion

Year	Author	Age	Sex	Side	Tumor size (mm)	Chief complaint	Disease control period	Preoperative diagnosis	Treatment	Prognosis
2005	Inagaki	51	F	L	30	Bulging	2 years	Inguinal hernia, fluid collection of cyst	Open mesh repair	No recurrence for 15 months
2005	Samson	79	M	R	n.p	Bulging and pain	6 months	Inguinal hernia	Open mesh repair	No recurrence for 18 months
2006	Ng	72	M	R	n.p	Distending discomfort	1 year	Inguinal hernia	Herniotomy	Open rt. hemicolectomy 3 months later
2008	Imazu	36	F	R	90 × 40	Bulging and pain	5 years	Inguinal hernia	Open mesh repair	No recurrence for 18 months
2009	Kubota	40s	F	R	n.p	Bulging and pain	8 months	Inguinal hernia	Open mesh repair	No recurrence for 18 months
2009	Takemoto	47	F	L	60 × 32	Bulging and pain	1 month	Inguinal hernia or soft tissue tumor or lymphadenopathy	Tumor resection	No recurrence for 12 months
2018	Yokota	53	F	R	90 × 40	Bulging	5 years	Inguinal hernia or neurogenic tumor or lymphocele	Open mesh repair	No recurrence for 24 months

BMPM is usually located in the pelvis. However, it can be found in the kidneys, bladder, lymph nodes, liver, and spleen. BMPM is often accompanied by a few symptoms when the tumor is small. However, once the tumor enlarges and exerts pressure on other organs, symptoms begin to appear. Most of the symptoms are non-specific, such as abdominal pain, palpable masses, and constipation. Given that the inguinal canal has a smaller space compared to the intra-abdominal cavity and is close to the body surface, the tumor may be detected at relatively smaller sizes. This suggests the possibility of early detection and treatment of BMPM arising from the inguinal canal. In the current case, the BMPM occurring at the inguinal region presented with a palpable mass, which allowed the patient to visit our hospital earlier when the masses were still relatively small.

In females, benign diseases, such as hydrocele of Nuck’s canal, ectopic endometriosis, lymphangioma, or adenomatoid tumor and malignant diseases, such as malignant mesothelioma or pseudomyxoma peritonei, can cause inguinal cystic tumors. In this case, we differentiated Nuck’s canal hydrocele as the main differential diagnosis due to the lack of menstruation-associated tumor growth and shrinkage as well as its epidemiological frequency. However, it is difficult to make a correct differential diagnosis based on the preoperative imaging examination of the tumor. Ultrasonography (US) is frequently the first examination requested because of its accessibility and lack of an irradiation source. On US, there are multicystic anechoic cysts with liquid content [16]. When the lesion contains a low-density, multi-loculated, thin-walled multicystic mass, CT is used to assess the location and extent of the cystic mass [17]. The best imaging technique is magnetic resonance imaging. On T1, BMPM appears as hypointense lesions, whereas on T2, they appear as hyper to intermediate intense lesions with mild wall contract enhancement [18]. Alternatively, surgical resection is often performed for diagnostic treatment; in the current case, tumor resection and hernia repair were performed despite the absence of any definite diagnosis of malignancy. Following pathological examination, a definitive diagnosis of BMPM was made, demonstrating that the cystic lesions were lined by a single layer of flattened or cuboidal regular mesothelial cells [19]. Immunostaining can also be helpful for a definitive diagnosis. In addition, a series of markers, such as cytokeratin 5/6, calretinin [5], and D2-40, have been reported by immunohistochemical analysis [20]. In particular, the D2-40 stain can strongly support a diagnosis of BMPM, associated with a specificity for mesothelial cells [21].

In general, reports have shown that abdominal BMPM has a recurrence rate of approximately 50% after surgical resection [22]. Thus, complete surgical resection seems to be an appropriate option for these patients. However, the optimal treatment for inguinal BMPM remains unclear. As the current patient was symptomatic and the differentiating BMPM from a hydrocele of Nuck’s canal and inguinal hernia was difficult, we decided to perform surgical resection for diagnostic and therapeutic purposes. Owing to its rarity, the recommended postoperative follow-up for inguinal BMPM is still unclear. Existing reports have shown varying durations for follow-up, with some cases being followed up after only a few months. In the current case, BMPM was found in the contralateral inguinal region 5 years after the first surgery. Regular follow-up may be necessary with BMPM occurring in the abdominal cavity. Moreover, our study highlights the need for accumulating more inguinal BMPM cases in the future.

## Conclusions

Here we report a case of BMPM occurring metachronously in bilateral inguinal canals. Although the pathogenesis of BMPM is still unclear, reactive changes may have contributed to the development of tumors in the groin. Surgical resection is the standard treatment of choice for BMPM. Appropriate postoperative follow-up for inguinal BMPM is yet to be established, and the accumulation of more inguinal BMPM cases is required.

## Data Availability

All data supporting this article are included in this manuscript.

## Abbreviations

BMPM: Benign multicystic peritoneal mesothelioma
CT: Computed tomography
US: Ultrasonography
